# Protein Hydrolysate from *Spirulina platensis* Prevents Dexamethasone-Induced Muscle Atrophy via Akt/Foxo3 Signaling in C2C12 Myotubes

**DOI:** 10.3390/md20060365

**Published:** 2022-05-29

**Authors:** Chi-Woo Lee, Yeok Boo Chang, Chun Woong Park, Sung Hee Han, Hyung Joo Suh, Yejin Ahn

**Affiliations:** 1 Department of Integrated Biomedical and Life Science, Graduate School, Korea University, Seoul 02841, Korea; beastory@korea.ac.kr (C.-W.L.); fjuae4@korea.ac.kr (Y.B.C.); woong9405@korea.ac.kr (C.W.P.); suh1960@korea.ac.kr (H.J.S.); 2 BK21FOUR R&E Center for Learning Health Systems, Korea University, Seoul 02841, Korea; 3 Institute of Human Behavior & Genetic, College of Medicine, Korea University, Seoul 02841, Korea; sungheeh@korea.ac.kr

**Keywords:** *Spirulina*, Collupulin, muscle atrophy, dexamethasone

## Abstract

Loss of muscle mass is the primary symptom of sarcopenia. Protein intake is recommended to prevent muscle mass loss, and *Spirulina platensis*, a microalga with high protein content, is a potential protein supplement. Here, we evaluated the differentiation ability of C2C12 cells and the inhibitory effect of *Spirulina* hydrolysates (SPH) prepared by Collupulin on dexamethasone (DEX)-treated C2C12 cells. SPH contained 578.27 mg/g protein and 92.30 mg/g branched-chain amino acids. SPH increased C2C12 myotube length and diameter, likely owing to increased MyoD1 and Myf5 expression. Inhibition of increased Atrogin-1, MuRF-1, and FoxO3 expression by SPH in DEX-treated C2C12 cells suppressed DEX-induced muscle atrophy. Moreover, SPH inhibited the DEX-induced increase in cytosolic p-Akt protein expression and suppressed the increase in nuclear FoxO3a protein expression, thereby suppressing the increase in the protein expression of the ubiquitin-proteasome-related factors Atrogin-1 and MuRF-1, which are involved in muscle atrophy. SPH suppressed DEX-induced muscle atrophy by activating the Akt/FoxO3a pathway. SPH promoted C2C12 myoblast differentiation into myotubes and inhibited DEX-induced myotube atrophy by suppressing Atrogin-1 and MuRF-1 expression and regulating the FoxO3a transcription factor. Collectively, SPH can be used as a functional food to inhibit muscle atrophy and promote muscle regeneration.

## 1. Introduction

Sarcopenia refers to a decrease in skeletal muscle mass owing to a decrease in the number and area of muscle fibers seen with increasing age. This significantly impacts the performance of daily life functions, thereby limiting the ability of the elderly to lead independent lives [1,2]. Sarcopenia is an important risk factor for physical disability. Maintaining adequate skeletal muscle mass and strength is crucial for maintaining normal body functions [3]. An imbalance in protein metabolism leads to sarcopenia, although the exact contribution of each factor depends on the research model [2,4]. Metabolic factors can contribute to this imbalance, including changes in anabolic hormone levels, catabolic stimulation due to inflammation or disease, poor physical activity, and nutritional factors, such as insufficient protein intake [4]. Additionally, several intracellular changes are involved, including regulation of protein synthesis, protease activation, ubiquitin conjugation, and autophagy [5].

Although the exact mechanism related to muscle atrophy is obscure, the activation of the ubiquitin-proteasome pathway is believed to be the primary cause of increased proteolysis. Moreover, muscle RING-finger protein-1 (MuRF1) is expressed in skeletal muscles and is directly involved in muscle protein degradation [6,7]. In addition, the expression of myogenin and myogenic differentiation 1 (MyoD1), which are transcription factors involved in muscle differentiation, is reduced owing to the activation of specific genes during muscle atrophy [8,9].

Hence, there is an increasing demand for food ingredients and natural substances that can help in muscle development and enhancement and have relatively low side-effects. Substances with this potential activity include proteins and peptides present in protein hydrolysates [10]. Moreover, oyster hydrolysate [11], potato protein hydrolysate [12], and collagen hydrolysate [13] exhibit muscle atrophy-inhibitory effects. In particular, proteins and peptides of marine origin possess this potential biological activity. *Spirulina platensis*, a blue-green alga, has a high protein content (55–70%) and contains essential amino acids. Furthermore, *Spirulina* is rich in the phycobiliproteins phycoerythrin, allophycocyanin, and phycocyanin, which account for 60% of its total protein content. Phycocyanin in *Spirulina* is the key ingredient used in food, cosmetics, and pharmaceuticals [14]. In addition, phycocyanin is one of the pigment components present in *Spirulina* that shows various physiological activities, such as anti-inflammatory, antidiabetic, and hepatoprotective activities. It is also receiving attention as a potential substance with angiotensin-converting enzyme-inhibitory and antioxidant activities, in addition to affecting lipid metabolism. These pigment components are considered potent pharmacological and medicinal agents owing to their antioxidant capacity [15,16]. However, previous studies did not analyze the inhibitory effect of *Spirulina* hydrolysates on muscle atrophy. In the present study, we found that *Spirulina* hydrolysate (SPH) exerts an inhibitory effect on dexamethasone (DEX)-induced atrophy of C2C12 cell-derived myotubes.

## 2. Results

### 2.1. The SPH Composition and Effect on C2C12 Cell Viability

The crude protein content of raw *Spirulina* was 671.67 mg/g, that of the *Spirulina* water extract was 27.12 mg/g, and that of SPH, a hydrolysate prepared by collupulin, was 578.27 mg/g, which was equivalent to 86.1% of the protein content in raw *Spirulina* (Table 1). Regarding the total amino-acid content, the glutamic acid and aspartic acid contents were 115.24 mg/g and 71.25 mg/g in raw *Spirulina*, 90.70 mg/g and 49.74 mg/g in the water extract, and 94.73 mg/g and 50.57 mg/g in SPH, respectively (Table 1). The content of branched-chain amino acids (BCAAs), the muscle-forming amino acids, was 133.41 mg/g in raw *Spirulina*, 80.16 mg/g in the water extract, and 92.30 mg/g in SPH (Table 1). SPH showed higher crude protein, amino-acid, and phycocyanin contents than the water extract. The cell viability assay revealed that SPH did not affect the viability of C2C12 cells treated with 25–1000 μg/mL SPH for 24 h (Figure S1), indicating that, at a concentration less than 1000 μg/mL, SPH was not cytotoxic. Thus, subsequent experiments were conducted with 1000 μg/mL or lower SPH concentration. To evaluate the myoblast differentiation-promoting effect of SPH, SPH was treated during the 6 day differentiation induction period. It was confirmed that SPH did not affect cell viability when treated for 6 days at a concentration of 50–150 μg/mL (Figure S2).

### 2.2. Effect of SPH on C2C12 Myotube Length and Diameter

Undifferentiated myoblasts differentiate into mature myotubes [17,18]. To evaluate the effect of SPH on myoblast differentiation, changes in the myotube length and diameter were measured. Giemsa staining confirmed that the differentiation of myoblasts into myotubes increased as the differentiation duration increased (Figure S3).

**Figure 1:**
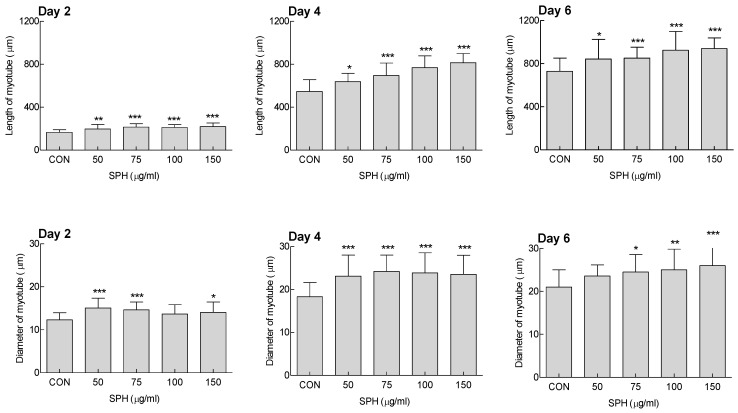
Effect of *Spirulina* hydrolysate (SPH) on length and diameter of C2C12 myotubes. Data are expressed as the mean ± standard deviation (SD). * *p* < 0.05, ** *p* < 0.01, and *** *p* < 0.001 vs. CON group (analysis of variance (ANOVA) followed by Tukey’s test). CON: control.

In addition, the myotubes tended to become longer and thicker with increasing differentiation duration (Figure 1). On day 6 of differentiation, 75, 100, and 150 μg/mL SPH significantly increased myotube diameter compared to the control group (*p* < 0.05, *p* < 0.01, and *p* < 0.001, respectively). Long and thick myotubes were formed when SPH concentration was greater than 75 μg/mL, which indicates that SPH can help promote myoblast differentiation.

### 2.3. Effect of SPH on C2C12 Myotube Differentiation-Related Factors

**Figure 2:**
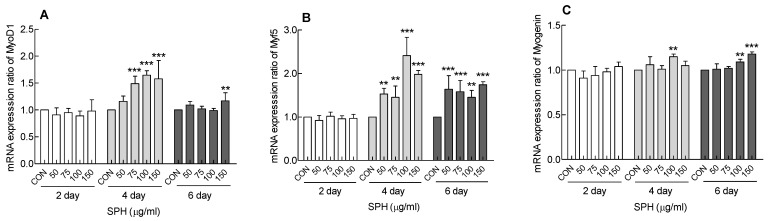
Effect of SPH on mRNA expression of (**A**) MyoD1, (**B**) Myf5, and (**C**) myogenin in C2C12 myotubes according to the differentiation duration. Data are expressed as the mean ± SD. ** *p* < 0.01, and *** *p* < 0.001 vs. CON group (ANOVA followed by Tukey’s test). CON: control, SPH: *Spirulina* hydrolysate, MyoD1: myogenic differentiation 1, Myf5: myogenic factor 5.

To evaluate the effect of SPH on C2C12 myotube differentiation and expression of related factors, the mRNA expression of MyoD1, myogenic factor 5 (Myf5), and myogenin was determined by real-time quantitative polymerase chain reaction (qPCR; Figure 2). SPH did not stimulate MyoD1 mRNA expression on day 2, whereas SPH (>75 μg/mL) significantly increased MyoD1 expression compared to the control group on day 4 (*p* < 0.001). On day 6, only 150 μg/mL SPH showed a significant difference compared to the control group (*p* < 0.01). Furthermore, SPH effectively increased MyoD1 expression at the mid-stage (day 4) of myotube differentiation. There was no difference in Myf5 expression at the initial stage of differentiation (day 2) among the groups. At the middle (day 4) and late (day 6) stages of differentiation, SPH enhanced Myf5 expression compared to the control group (*p* < 0.01 and *p* < 0.001, respectively). Similarly, myogenin expression was significantly higher in the 100 and 150 μg/mL SPH groups than in the control group on day 6 of differentiation (*p* < 0.01 and *p* < 0.001, respectively). Collectively, SPH contributed to the increase in myotube length and diameter by increasing the expression of MyoD1 and Myf5.

### 2.4. Effect of SPH on the Protein Expression of MyoD1 and Myogenin of C2C12 Myotubes at Day 6 of Differentiation

**Figure 3:**
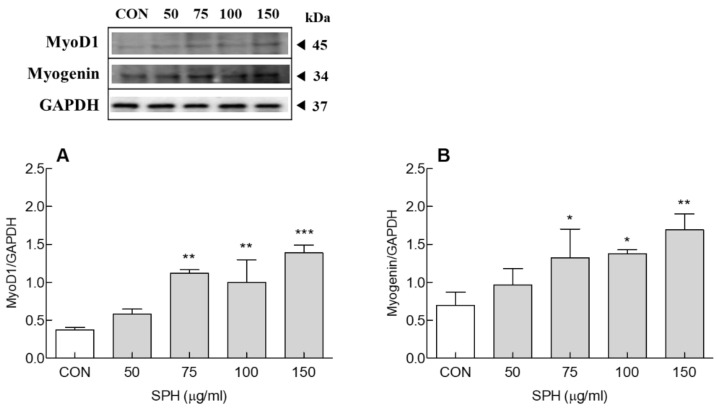
Effect of SPH on the protein expression of (**A**) MyoD1 and (**B**) myogenin in C2C12 myotubes. Cells were differentiated for 6 days with SPH treatment. Data are expressed as the mean ± SD. * *p* < 0.05, ** *p* < 0.01, and *** *p* < 0.001 vs. CON group (ANOVA followed by Tukey’s test). CON: control, SPH: *Spirulina* hydrolysate, MyoD1: myogenic differentiation 1.

The effect of SPH on the protein expression of myotube differentiation-related factors in the late stage of differentiation was confirmed by Western blotting (Figure 3). On day 6 of differentiation, MyoD1 protein expression was significantly increased by SPH treatment (>75 μg/mL) compared to the CON group (*p* < 0.01 and *p* < 0.001; Figure 3A). In addition, SPH treatment (>75 µg/mL) significantly increased myogenin expression compared to the CON group (*p* < 0.05 and *p* < 0.01, Figure 3B). Therefore, SPH (150 μg/mL) treatment promoted muscle differentiation by significantly increasing the mRNA and protein expression of muscle differentiation factors such as MyoD1 and myogenin in the late stage of differentiation.

### 2.5. Effect of SPH on Myotube Length and Diameter in DEX-Treated C2C12 Myotubes

**Figure 4:**
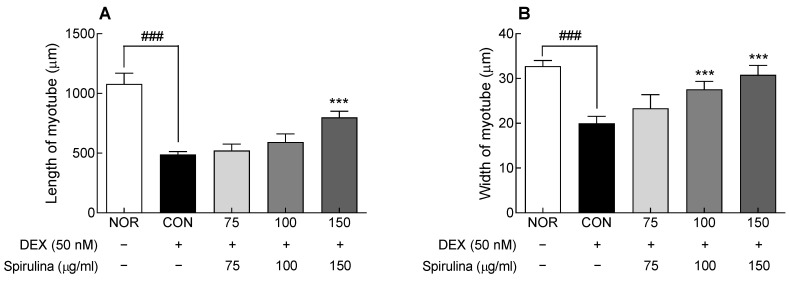
Effect of *Spirulina* hydrolysate (SPH) on (**A**) length and (**B**) diameter of C2C12 myotubes treated with dexamethasone (DEX; 50 nM). Data are expressed as the mean ± SD. *** *p* < 0.001 vs. CON group. ^###^ *p* < 0.001 for CON group vs. NOR group (ANOVA followed by Tukey’s test). NOR; normal, CON: control, SPH: *Spirulina* hydrolysate, DEX: dexamethasone.

When muscle atrophy is induced, the number and diameter of muscle fibers decrease through the breakdown of muscle proteins, resulting in a decrease in total muscle mass [19,20]. To evaluate the protective effect of SPH on muscle atrophy, the length and thickness of muscle fibers were analyzed by Jenner–Giemsa staining (Figure S5). In the CON group treated only with DEX, the length and width of the myotube were significantly reduced compared to the NOR group (*p* < 0.001; Figure 4A,B), confirming that DEX-induced muscle atrophy was effectively induced. In contrast, SPH (150 μg/mL) treatment significantly increased muscle fiber length and thickness compared to the CON group (*p* < 0.001 and *p* < 0.01, respectively; Figure 4A,B). Myotube degradation was inhibited by SPH treatment, indicating that SPH could protect against DEX-induced muscle atrophy.

### 2.6. Effects of SPH on the Expression of Atrogin-1, MuRF-1, and Forkhead Box O3a (FoxO3a) in DEX-Treated C2C12 Myotubes

**Figure 5:**
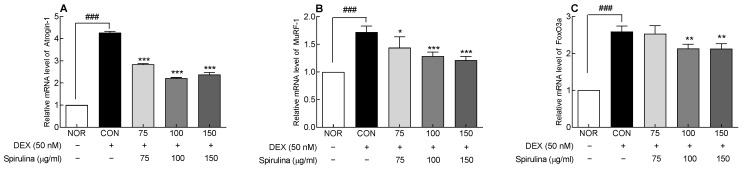
Effect of SPH on mRNA expression of (**A**) Atrogin-1, (**B**) MuRF-1, and (**C**) FoxO3a in C2C12 myotubes treated with dexamethasone (DEX; 50 nM). Data are expressed as the mean ± SD. * *p* < 0.05, ** *p* < 0.01, and *** *p* < 0.001 vs. CON group. ^###^ *p* < 0.001 for CON group vs. NOR group (ANOVA followed by Tukey’s test). NOR: normal, CON: control, SPH: *Spirulina* hydrolysate, DEX: dexamethasone, Atrogin-1: atrogin-1/a muscle-specific F-box protein, Murf-1: muscle RING-finger protein-1, FoxO3a: forkhead box O3a.

The effect of SPH on DEX-induced muscle atrophy was evaluated by measuring the mRNA expression of Atrogin-1 and MuRF1, muscle-specific ubiquitin ligases (Figure 5A,B). The mRNA expression of Atrogin-1 and MuRF-1 was significantly higher (*p* < 0.001) in the control group (cells treated with 50 nM DEX) compared to the normal group. In contrast, SPH and DEX cotreatment suppressed the DEX-induced increase in Atrogin-1 and MuRF-1 expression, and a significant inhibitory effect of SPH was revealed at 75–150 μg/mL concentrations, compared to the control group (*p* < 0.05 and *p* < 0.001, respectively).

FoxO3a is a nuclear factor that regulates muscle atrophy and plays a role in regulating Atrogin-1 and MuRF-1 expression, which promote protein degradation in skeletal muscles. FoxO3a expression was significantly higher in the control group compared to the normal group (*p* < 0.001; Figure 5C). However, SPH decreased Foxo3a expression, which was increased by DEX, in a dose-dependent manner. In particular, 100 and 150 μg/mL SPH significantly lowered FoxO3a expression compared to the control group (*p* < 0.01). These results suggest that SPH suppressed the DEX-induced increase in the expression of muscle atrophy factors.

### 2.7. Effect of SPH on the Protein Expression of Cytosolic Akt and Nuclear Atrogin-1, MuRF-1, and FoxO3a in DEX-Treated C2C12 Myotubes

**Figure 6:**
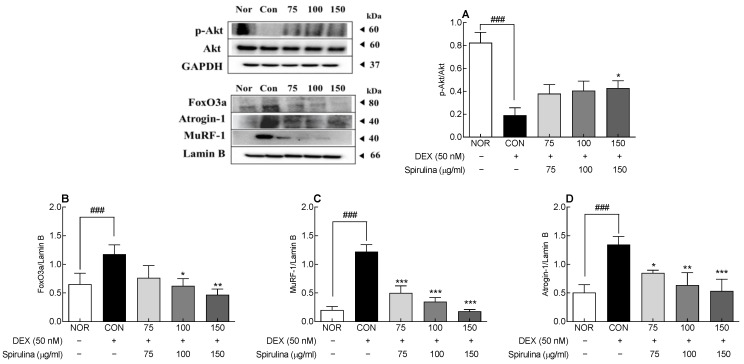
Effect of SPH on the protein expression of (**A**) cytosolic Akt and nuclear (**B**) FoxO3a, (**C**) MuRF-1, and (**D**) Atrogin-1 in C2C12 myotubes treated with DEX (50 nM). Data are expressed as the mean ± SD. * *p* < 0.05, ** *p* < 0.01, and *** *p* < 0.001 vs. CON group. *^###^ p* < 0.001 for CON group vs. NOR group (ANOVA followed by Tukey’s test). NOR: normal, CON: control, SPH: *Spirulina* hydrolysate, DEX: dexamethasone, Akt: protein kinase B, Atrogin-1: Atrogin-1/a muscle-specific F-box protein, Murf-1: muscle RING-finger protein-1, FoxO3a: forkhead box O3a.

To investigate the mechanism underlying the inhibitory effect of SPH on muscle atrophy, proteins involved in the cytoplasmic and nuclear signaling pathways were assessed by Western blotting (Figure 6). Akt, a major factor in signaling pathways related to protein metabolism, is involved in muscle atrophy. DEX significantly reduced Akt protein phosphorylation in the control group compared to the normal group (*p* < 0.001; Figure 6A). Contrarily, SPH increased Akt protein phosphorylation, which was reduced by DEX, in a dose-dependent manner. In fact, 150 μg/mL SPH significantly increased p-Akt protein levels compared to the control group (*p* < 0.05).

Furthermore, DEX significantly increased the expression of nuclear muscle atrophy-related proteins, such as Atrogin-1, MuRF-1, and FoxO3a, compared to the normal group (*p* < 0.001; Figure 6B–D). However, SPH inhibited the increase in the expression of nucleoproteins involved in muscle atrophy in a concentration-dependent manner. The increase in Atrogin-1 and MuRF-1 protein expression was significantly inhibited by SPH at all concentrations (75–150 μg/mL; *p* < 0.05, *p* < 0.01, and *p* < 0.001, respectively). Similarly, 100 and 150 μg/mL SPH significantly inhibited the increase in FoxO3a protein expression compared to the control group (*p* < 0.05 and *p* < 0.01, respectively). SPH suppressed the DEX-induced increase in mRNA and protein expression of Atrogin-1, MuRF-1, and FoxO3a; hence, it is a potential therapeutic agent to suppress muscle atrophy.

## 3. Discussion

Muscle atrophy, along with malnutrition and decreased physical activity, is commonly observed in chronic diseases, and several intracellular changes, such as protein synthesis regulation, protease activation, ubiquitin conjugation, and autophagy, are known to be involved [21]. The increase in muscle differentiation capacity and muscle inhibition minimize the loss of muscle mass, along with protein-rich nutrition and physical exercise [22]. Hydrolysates composed of active peptides supply proteins that are easily absorbed and can prevent age-related diseases. Hydrolysates of microalgal proteins, such as *Chlorella vulgaris*, *Dunaliella salina*, and *S. platensis*, have high utility value as active peptides [23]. The hydrolysate of *Spirulina* shows antioxidant [24], anti-inflammatory [25], anticancer [26], and immune-enhancing activities [25]. In this study, the SPH prepared by Collupulin contained 578.27 mg/g protein, 2.09 mg/g C-phycocyanin, and 2.19 mg/g allophycocyanin (Table 1). Because microalgae are cultured in an environment exposed to high oxidative stress, they produce pigments with antioxidant activity, such as chlorophyll, carotene, and phycobiliprotein, making them beneficial for health. BCAAs are involved in muscle protein synthesis, and, among BCAAs, leucine intake reportedly inhibits muscle loss by suppressing Atrogin-1 expression and autophagy [27,28]. Here, SPH contained 92.30 mg/g BCAAs; therefore, SPH may be a useful protein source to inhibit muscle atrophy.

Myogenesis (myogenic differentiation) is a necessary process in muscle regeneration. In this process, several myoblasts fuse and differentiate into myotubes, which are multinucleated tubular cells [29]. MyoD and Myf-5 are classified as primary myogenic regulators and play a role in inducing myoblasts, whereas myogenin, Myf-6, and myocyte enhancer factor 2, which are the secondary myogenic regulators, play a role in differentiating myoblasts into myotubular cells [18]. In this study, SPH increased the expression of the representative myogenic factors MyoD1, Myf5, and myogenin (Figure 2). Additionally, SPH seemed to significantly contribute to the increase in MyoD1 and Myrf5 expression. The SPH-mediated increase in the mRNA expression of myogenic factors appeared to be involved in the formation of long and thick myotubes (Figure 1 and Figure S2), suggesting that SPH promotes C2C12 myotube differentiation.

Chronic diseases and aging cause muscle protein imbalance, and an increase in proteolysis via activation of the ubiquitin-proteasome pathway is considered to be involved in this process. Atrogin-1 and MuRF1 are the major ubiquitin ligases directly involved in muscle protein degradation [30,31]. DEX, a synthetic glucocorticoid, increases the rate of proteolysis in muscles and induces muscle atrophy in vivo and in vitro [32]. In a muscle atrophy model, the expression of Atrogin-1 and MuRF1 increased, and that of muscle differentiation factors, such as myogenin and MyoD1, decreased [33]. MuRF-1 mRNA expression was significantly increased due to DEX (25-100 nM) treatment, but DEX (>75 nM) showed low cell viability (Figure S4); thus, the concentration of DEX inducing muscle atrophy was set to 50 nM.

In the present study, SPH suppressed the DEX-induced increase in the mRNA and protein expression of Atrogin-1 and MuRF1 (Figure 3A,B and Figure 4C,D). The DEX-induced increase in the mRNA and protein expression of FoxO3a, a muscle-specific transcription factor in the nucleus, was also suppressed by SPH (Figure 3C and Figure 4B). FoxO3a maintains cell survival by regulating the cell cycle, differentiation, and proliferation. However, sustained FoxO3a activation leads to proteolysis and muscle atrophy [34]. Inhibition of Foxo3a activation suppresses Atrogin-1 induction and DEX-induced muscular atrophy. Akt phosphorylation inhibits muscle atrophy by blocking the nuclear translocation of FoxO3, an important downstream target protein of the PI3K/Akt signaling pathway [35]. Blocking the nuclear translocation of FoxO inhibits FoxO-dependent gene activation. In turn, decreased nuclear FoxO protein expression reduces Atrogin-1 and MuRF1 protein levels and inhibits proteolysis [36]. Here, SPH inhibited the DEX-induced decrease in Akt phosphorylation (Figure 4A).

In conclusion, the inhibitory effect of SPH on muscle atrophy was attributed to the suppression of the DEX-induced increase in the expression of muscle atrophy-related genes and proteins in C2C12 myotubes. In particular, the inhibitory effect of SPH on muscle atrophy was apparently due to the increase in the expression of genes encoding muscle differentiation factors (MyoD1, Myf5, and myogenin) and downregulation of the induced gene and protein expression of muscle atrophy factors (Atrogin-1, MuRF-1, and FoxO3a). SPH showed an inhibitory effect on muscle atrophy via activating the Akt/FoxO3a signaling pathway. Future studies should elucidate the efficacy and mechanism of SPH in inhibiting muscle atrophy using animal models to develop SPH as a health functional food material for the prevention, treatment, and improvement of muscle atrophy.

## 4. Materials and Methods

### 4.1. Materials

*Spirulina* powders were purchased from Earthrise Nutritional LLC (Irvine, CA, USA). *Spirulina* powder was dissolved in 0.05 M citric acid buffer (pH 5.0, *w*/*v* = 1:20), and Collupulin (DSM Food Specialties, MA Delft, the Netherlands) was added at 3% by weight of the substrate. After 8 h of enzymatic reaction (50 °C, 120 rpm), the enzyme was inactivated (95 °C, 15 min), and the supernatant (SPH) was collected by centrifugation (5000 rpm, 20 min, 4 °C). SPH was lyophilized and stored at −20 °C until the experiment. The crude protein and amino-acid content of SPH was measured using an automatic amino-acid analyzer (Biochrom 20, Pharmacia-Biotech, Freiburg, Germany) [37]. Dulbecco’s modified Eagle medium (DMEM), fetal bovine serum (FBS), horse serum (HS), and penicillin/streptomycin (PS) were purchased from WELGENE (Daegu, Korea). DEX and water-soluble tetrazolium salt-8 (WST-8) assay kits were purchased from Sigma-Aldrich Chemical Co., Ltd. (St. Louis, MO, USA) and Biomax (Seoul, Korea), respectively. Primary antibodies against MyoD1 (1:500, sc-12732, SantaCruz, Dallas, TX, USA), Myogenin (1:500, sc-377460, SantaCruz), MAFbx/Atrogin-1 (1:1000, ab168372, Abcam, Cambridge, MA, USA), MuRF1 (1:1000, PA5-76695, Invitrogen, Carlsbad, CA, USA), Akt (1:1000, #9272, Cell Signaling Technology, Beverly, MA, USA), p-Akt (1:1000, #9271, Cell Signaling Technology), FoxO3α (1:1000, #2497, Cell Signaling Technology), p-FoxO3α (1:1000, #9466, Cell Signaling Technology), GAPDH (1:1000, #5174, Cell Signaling Technology), and Lamin B1 (1:1000, #12586, Cell Signaling Technology) were used for protein analysis. Horseradish peroxidase-linked anti-rabbit IgG secondary antibodies (1:2000, #7074) were obtained from Cell Signaling Technology.

### 4.2. Myotube Differentiation and DEX-Induced Muscle Atrophy of C2C12 Cells

The C2C12 cell line (CRL-1772) was obtained from ATCC (LGC Promochem, Teddington, UK). C2C12 myoblasts (3 × 10^5^ cells/well) were cultured in six-well culture plates with growth medium (DMEM containing 10% FBS and 1% PS) for 24 h. When the cells reached 100% confluence, the medium was replaced with a differentiation medium (DMEM containing 2% HS and 1% PS) to induce myotube differentiation for 6 days. The differentiation medium was changed every 2 days, and myoblasts were differentiated into complete myotubes for 6 days. DEX was used to induce muscle atrophy in C2C12 cells. DEX, a synthetic glucocorticoid, is known to induce muscle atrophy by increasing proteolysis through modulation of the ubiquitin-proteasome pathway [36]. To induce muscle atrophy, fully differentiated C2C12 cells were treated with DEX (50 nM) for 48 h for 6 days. SPH was treated with DEX diluted in DMEM medium (containing 0.5% HS and 1% PS), and DEX and SPH were freshly supplied every 24 h.

### 4.3. Cell Viability

The viability of C2C12 myoblasts treated with SPH was confirmed using the WST-8 assay. Briefly, cells were cultured in a 96-well plate (1 × 10^5^ cells/well) in the growth medium and maintained at 5% CO_2_ and 37 °C. After 24 h, SPH was added at various concentrations for 24 h or 6 days, and then 10 μL of WST-8 reagent was added. After 1 h, the absorbance was measured at 450 nm. Cell viability was calculated as the percentage of the control (0 μg/mL): A450 of SPH-treated cells/A450 of control cells × 100.

### 4.4. Measurement of Myotube Length and Diameter

Myotube length and diameter were measured using images of Jenner–Giemsa-stained C2C12 cells [38]. Cultured cells were washed with phosphate-buffered saline (PBS), fixed with 100% methanol for 5 min, dried for 10 min, diluted three times with sodium phosphate solution (1 mM PBS, pH 5.6) and Jenner’s staining solution (Sigma-Aldrich Inc.), and incubated for 5 min. After washing with PBS, the cells were incubated with 1 mL of 20-fold diluted Giemsa stain at 25 °C for 10 min, and then washed three times with PBS to determine the morphological changes of C2C12 cells. To evaluate the shape change, images were observed at a magnification of 200× using an inverted microscope, and pictures were captured using the Axio Vision program (Carl Zeiss, Oberkochen, Germany). Myotube length and diameter were measured and quantified using the ImageJ software (Scion, Frederick, MD, USA).

### 4.5. qPCR

Quantification of mRNA expression was performed as described in previous methods [32] using the SYBR™ Green PCR Master Mix (Applied Biosystems, Foster City, CA, USA) and StepOne™ Real-Time PCR System (Applied Biosystems). The target mRNA expression was analyzed by relative quantification using the CT value of glyceraldehyde 3-phosphate dehydrogenase (GAPDH, NM_001289726.1), a widely used housekeeping gene. The target genes analyzed were MyoD1 (NM_010866.2), Myf5 (NM_008656.5), myogenin (NM_031189.2), Atrogin-1/a muscle-specific F-box protein (Atrogin-1, NM_026346.3)**,** Murf-1 (NM_001039048.2), and FoxO3a (NM_001376967.1).

### 4.6. Western Blot Analysis

The prepared cells were lysed according to a previous method [39] using a lysis buffer containing 250 mM NaCl, 25 mM Tris-HCl (pH 7.5), 10 mM ethylenediaminetetraacetic acid, 1% NP-40, 0.1 mM phenyl-methyl sulfonyl fluoride, and protease inhibitors. The cell lysate was centrifuged at 13,000× *g* and 5 °C for 20 min to remove debris. Nuclear fractions were isolated using NE-PER™ nuclear and cytoplasmic extraction reagents (Thermo Fisher Scientific Inc., Rockford, IL, USA). After protein quantification, an equal amount of protein was separated by sodium dodecyl sulfate polyacrylamide gel electrophoresis (SDS-PAGE) and transferred to a nitrocellulose membrane (Schleicher & Schuell GmbH, Keene, NH, USA). The membrane was probed using an appropriate antibody and enhanced chemiluminescence (Amersham Biosciences Corp., Piscataway, NJ, USA) solution to determine the protein expression. The band of each protein was quantified as the expression ratio compared to the expression of GAPDH (cytosolic protein) and Lamin B1 (nuclear protein).

### 4.7. Statistical Analysis

Experimental results are expressed as the mean ± standard deviation. Analysis of variance was performed to test the significance of the experimental results, and the results were verified at *p* < 0.05 using Tukey’s multiple range test. Statistical analysis was performed using the SPSS statistical program (SPSS12, SPSS Inc., Chicago, IL, USA).

## Figures and Tables

**Table 1:** Crude protein, phycocyanin, and total amino-acid contents of raw *Spirulina*, *Spirulina* hydrolysate, and the water extract of *Spirulina*.

mg/g		*Spirulina*	*Spirulina* Extract	
			Water Extract	Enzyme Hydrolysis
Crude protein		671.67 ± 5.76	27.12± 1.52	578.27 ± 4.68
C-phycocyanin			0.68 ± 0.002	2.09 ± 0.02
Allophycocyanin			0.47 ± 0.001	2.19 ± 0.01
Amino acids	Aspartic acid	71.25 ± 2.98	49.73 ± 1.29	50.57 ± 2.48
	Glutamic acid	115.24 ± 8.98	90.69 ± 1.08	94.73 ± 2.47
	Serine	35.88 ± 0.58	22.40 ± 1.02	24.09 ± 1.08
	Histidine	10.81 ± 1.89	4.56 ± 0.17	6.84 ± 0.36
	Glycine	38.59 ± 0.82	24.39 ± 0.31	23.14 ± 0.73
	Threonine	36.55 ± 1.34	24.21 ± 0.49	27.62 ± 0.93
	Arginine	51.39 ± 1.04	31.93 ± 1.18	30.27 ± 0.87
	Alanine	54.18 ± 1.98	42.86 ± 1.07	37.54 ± 1.58
	Tyrosine	28.59 ± 1.16	19.26 ± 0.87	16.09 ± 0.32
	Valine	38.58 ± 0.73	24.76 ± 1.19	28.61 ± 0.73
	Methionine	17.11 ± 0.33	11.32 ± 0.76	12.04 ± 0.39
	Phenylalanine	33.61 ± 1.06	16.94 ± 0.58	20.70 ± 0.80
	Isoleucine	36.45 ± 0.73	24.27 ± 0.79	25.97 ± 0.78
	Leucine	58.38 ± 1.99	31.12 ± 1.03	37.71 ± 1.16
	Lysine	32.47 ± 1.25	15.93 ± 0.77	18.04 ± 0.66
	Proline	14.72 ± 0.37	7.755 ± 0.53	9.25 ± 0.58
	BCAA	133.41 ± 3.45	80.16 ± 3.01	92.30 ± 2.67

Data are expressed as the mean ± standard deviation. BCAA: branched-chain amino acid.

## Data Availability

The data are available within the article.

## Supplementary Materials

The following supporting information can be downloaded at https://www.mdpi.com/article/10.3390/md20060365/s1: Figure S1. Cell viability of *Spirulina* hydrolysate (SPH)-treated C2C12 cells. C2C12 cells were treated with various concentrations of SPH for 24 h. Data are expressed as the mean ± standard deviation of three independent experiments. ns: not significant compared to CON group (ANOVA followed by Tukey’s test). CON: control (0 μg/mL), SPH: *Spirulina* hydrolysate; Figure S2. Cell viability of *Spirulina* hydrolysate (SPH)-treated C2C12 cells. C2C12 cells were treated with various concentrations of SPH for 6 days. Data are expressed as the mean ± standard deviation of three independent experiments. ns: not significant compared to CON group (ANOVA followed by Tukey’s test). CON: control (0 μg/mL), SPH: *Spirulina* hydrolysate; Figure S3. Photographs of C2C12 myotubes treated with SPH. Jenner–Giemsa staining was performed on days 2, 4, and 6 of C2C12 cell differentiation; Figure S4. Effect of dexamethasone on (**A**) cell viability and (**B**) relative mRNA expression in C2C12 myotubes. C2C12 cells were treated with various concentrations of dexamethasone to evaluate cell viability by WST-8 analysis and MuRF-1 mRNA expression by qPCR. C2C12 cells were fully differentiated for 6 days and then exposed to dexamethasone for 48 h. Data are presented as the mean ± SD. **** p* < 0.001 vs. CON group (ANOVA followed by Tukey’s test). CON: control (0 μg/mL), Murf-1: muscle RING-finger protein-1; Figure S5. Photographs of C2C12 myotubes treated with SPH in DEX (50 nM)-induced muscle atrophy model. Jenner–Giemsa staining was performed 48 h after DEX treatment.
