# Supplementary material for: Protein Hydrolysate from Spirulina platensis Prevents Dexamethasone-Induced Muscle Atrophy via Akt/Foxo3 Signaling in C2C12 Myotubes
Source: Mar Drugs. 2022 May 29;20(6):365. doi: 10.3390/md20060365 (PMC9229963; doi:10.3390/md20060365)
Supplement: Supplementary file 1 [file marinedrugs-20-00365-s001.zip › marinedrugs-1691537-supplementary.pdf]

Supplementary data

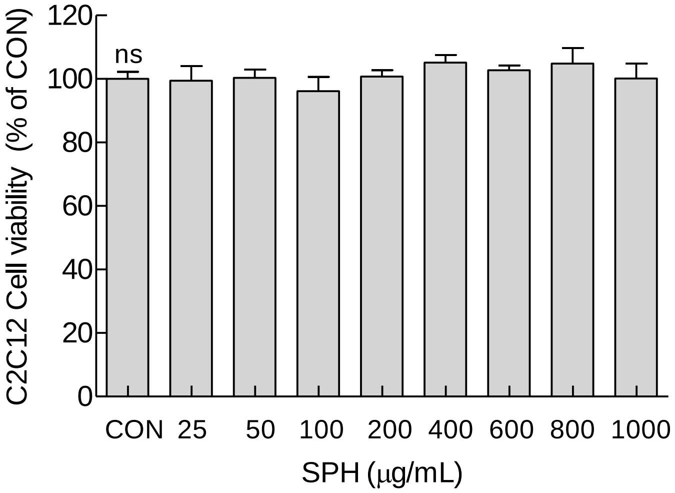

Figure S1. Cell viability of *Spirulina* hydrolysate (SPH)-treated C2C12 cells. C2C12 cells were treated with various concentrations of SPH for 24 h. Data are expressed as the mean ± standard deviation of three independent experiments. ns: not significant compared to CON group (ANOVA followed by Tukey's test). CON: control (0 μg/mL), SPH: *Spirulina* hydrolysate

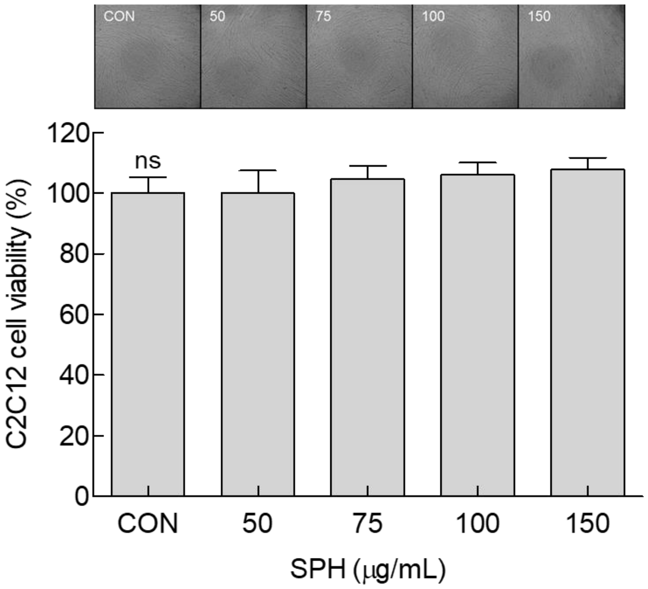

Figure S2. Cell viability of *Spirulina* hydrolysate (SPH)-treated C2C12 cells. C2C12 cells were treated with various concentrations of SPH for 6 days. Data are expressed as the mean ± standard deviation of three independent experiments. ns: not significant compared to CON group (ANOVA followed by Tukey's test). CON: control (0 μg/mL), SPH: *Spirulina* hydrolysate

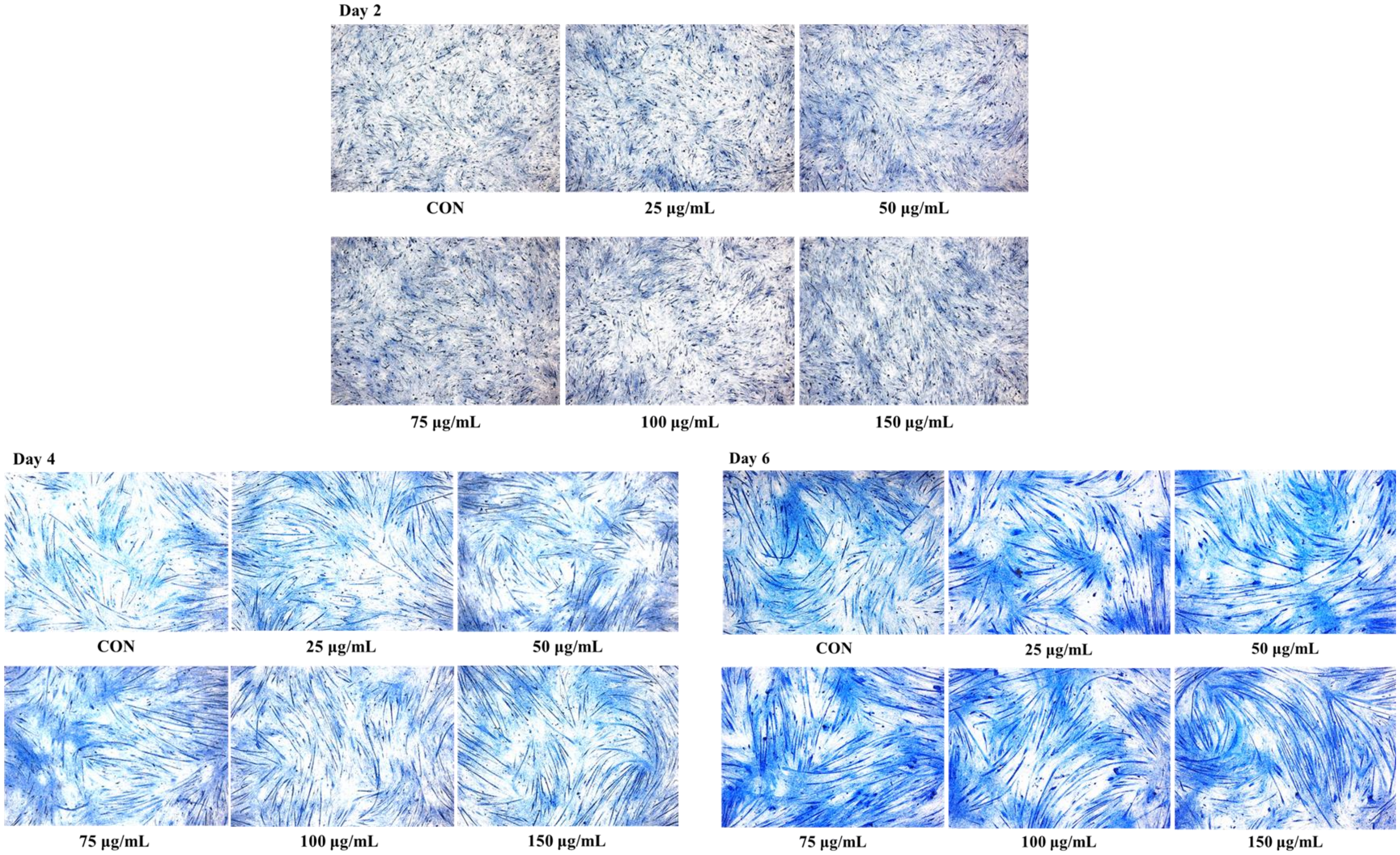

Figure S3. Photographs of C2C12 myotubes treated with SPH. Jenner–Giemsa staining was performed on days 2, 4, and 6 of C2C12 cell differentiation.

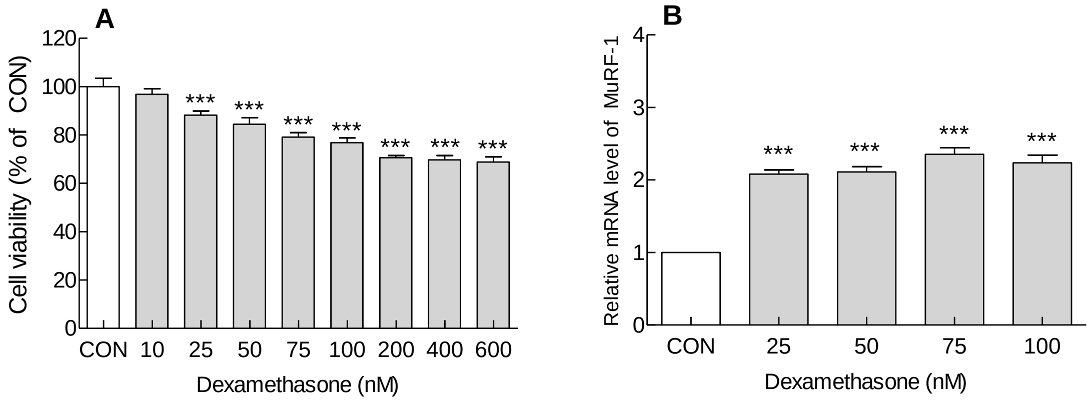

Figure S4. Effect of dexamethasone on (**A**) cell viability and (**B**) relative mRNA expression in C2C12 myotubes. C2C12 cells were treated with various concentrations of dexamethasone to evaluate cell viability by WST-8 analysis and MuRF-1 mRNA expression by qPCR. C2C12 cells were fully differentiated for 6 days and then exposed to dexamethasone for 48 h. Data are presented as the mean ± SD. *** $p < 0.001$ vs. CON group (ANOVA followed by Tukey's test). CON: control (0 μg/mL), Murf-1: muscle RING-finger protein-1.

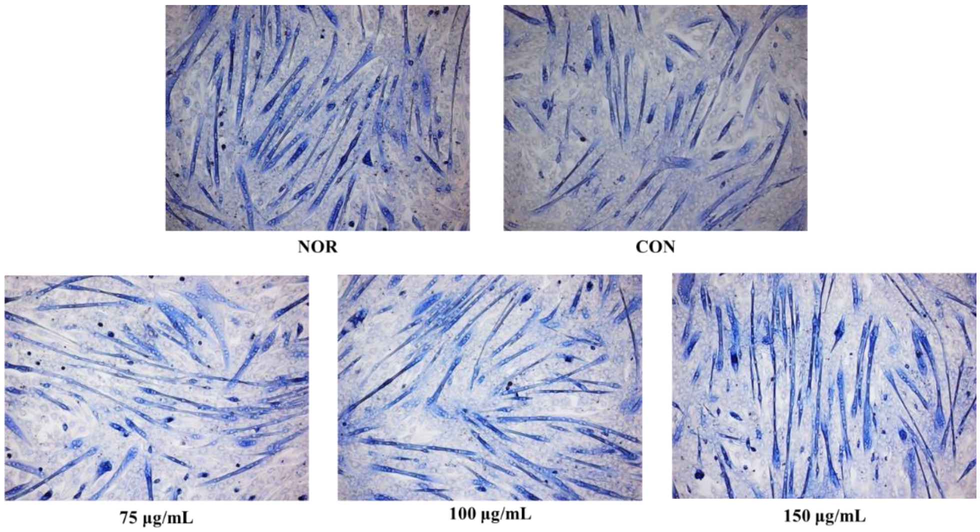

Figure S5. Photographs of C2C12 myotubes treated with SPH in DEX (50 nM)-induced muscle atrophy model. Jenner–Giemsa staining was performed 48 h after DEX treatment.
